# *E3 Siah* ubiquitin ligase regulates dichotomous spermatogenesis in *Sitotroga cerealella*

**DOI:** 10.3389/fcell.2024.1507725

**Published:** 2025-01-10

**Authors:** Sakhawat Shah, Chun-Mei Shi, Karam Khamis Elgizawy, Wen-Han Yan, Gang Wu, Xiao-Ping Wang, Feng-Lian Yang

**Affiliations:** ^1^ Hubei Key Laboratory of Insect Resources Utilization and Sustainable Pest Management, College of Plant Science and Technology, Huazhong Agricultural University, Wuhan, Hubei, China; ^2^ College of Horticulture and Forestry, Huazhong Agricultural University, Wuhan, China; ^3^ Plant Protection Department, Faculty of Agriculture, Benha University, Moshtohor, Toukh, Egypt

**Keywords:** dichotomous spermatogenesis, eupyrene, apyrene, *Sitotroga cerealella*, E3 ligase Siah

## Abstract

Spermatogenesis in Lepidoptera holds significant importance due to its unique process of dichotomous spermatogenesis, yielding eupyrene and apyrene spermatozoa through a complex molecular mechanism. While E3 ubiquitin ligases are known to play vital roles in spermatogenesis across various processes, their functions in dichotomous spermatogenesis remain less known. We utilized the RNAi, biochemical and microscopic procedures to unravel the function of *ScE3 Siah* in dichotomous spermatogenesis of adult *Sitotroga cerealella*. In *S*. *cerealella E3* ligase *Siah* predominantly expressed in adult tissues. Knockdown of *ScE3 Siah* leads to disruptions in testes and sperm morphology, affecting the structure of eupyrene and apyrene sperm bundles and causing defective ultrastructure in eupyrene sperm. This disruption results in a reduction in the number of dichotomous sperms and significantly reduces their motility. Moreover, *ScE3 Siah* knockdown inhibits the transfer and motility of dichotomous sperm, impacting spermatophore formation in females and ultimately reducing egg production. Understanding the role of *ScE3 Siah* is not only crucial for comprehending the complex processes involved in dichotomous spermatogenesis and fertilization but also provides an avenue for sustainable pest control management.

## Introduction

The ubiquitin proteasome system (UPS) is a crucial protein degradation pathway in eukaryotes, responsible for the degradation of approximately 75% of cellular proteins. The 26S proteasome, an ATP-dependent protein degradation molecular complex, encompasses over 30 distinct subunits with a total molecular mass exceeding 2,400 kDa (Glotzer et al., 1991). It consists of a 20S hollow cylindrical core particle (CP) and a 19S regulatory particle (RP) (Lander et al., 2012). The 20S CP is composed of four rings, each containing seven subunits. The outer two rings consist of subunits α1-7, while the inner two rings comprise subunits β1-7 (Seemuller et al., 1995). The CP subunits possess an active protease site and are thoroughly known for their proteolytic functions (Pathare et al., 2014; Lee et al., 2011). The 19S RP manage substrate recognition, deubiquitylation, unfolding, and the transfer of substrates into the 20S CP for proteolysis (Tomko and Hochstrasser, 2013; Finley, 2009).

The ubiquitination process comprises a hierarchical sequence of enzymatic actions carried out by E1, E2, and E3 enzymes. Firstly, E1, the ubiquitin activating enzyme, initiates by activating ubiquitin through ATP hydrolysis to ADP and binding it to the carboxyl end of ubiquitin via a thioester bond. Subsequently, E2, the ubiquitin-conjugating enzyme, passes the activated ubiquitin molecule to its cysteine residue. Finally, E3, the ubiquitin ligase enzyme, assists in transferring ubiquitin from E2 to the target substrate protein (Cruz Walma et al., 2022; Pla-Prats and Thomä, 2022).

Compared to E1 and E2 genes, E3 ligases are abundant. An analysis of the human genome using bioinformatics techniques identified the expression of 634 E3s, 41 E2s, and 8 E1s (Liu et al., 2019). Based on their binding domain structure and ubiquitin transfer mechanism, E3 ligases can be broadly classified into three groups: RING (*Really interesting new gene*), HECT (*homologous to the E6-associated protein carboxyl domain*), and RBR (*RING-between-RING*) (Cruz Walma et al., 2022; Deshaies and Joazeiro, 2009). The RING domain family constitutes the largest class of E3 ligases, accounting for half of all human E3 ligases and being responsible for 20% of cellular ubiquitination (Liu et al., 2019; Baek et al., 2020).

UPS present in both mammalian and non-mammalian spermatozoa play a critical role in regulating key events of fertilization, such as sperm penetration of the vitelline layer (Sutovsky, 2011). Studies using whole-body and tissue extracts from mice have shown that the rate of ubiquitination in the testis is four times higher than in other tissues (Rajapurohitam et al., 2002). The UPS is essential for various stages of spermatogenesis, including meiotic and mitotic division and recombination, sperm motility, sperm capacitation, and sperm-egg penetration (Sutovsky, 2011). It is also vital for spermatogonial development, meiotic sex chromosome inactivation, modulation of spermatogenesis, nucleosome remodeling, acrosome and sperm tail biogenesis, and spermatid maturation (Rajapurohitam et al., 2002). The characterization of sperm proteomes in *Drosophila* and *Manduca sexta* has revealed the presence of proteasome complex subunits, which are necessary for spermatogenesis individualization and nuclear maturation (Zhong et al., 2007; Whittington et al., 2015). The involvement of different E3 ligases in spermatogenesis regulation, including spermatid individualization, regulation of junctional complexes, spermatocyte meiosis, and germ cell apoptosis has been reported (Richburg et al., 2014), warranting extensive further research into this field.

One specific E3 ligase of interest is *Siah* (*seven in absentia homologue*), which contains a RING domain and is highly conserved in mammals. *Siah* controls the degradation of proteins such as β-catenin, N-CoR, and DCC (Dickins et al., 2002). Mutation of *Siah1a* in mammals has been shown to cause infertility due to failure in the progression of meiosis I (Dickins et al., 2002). However, less is known about the role of *E3 Siah* ligase in dichotomous spermatogenesis. Dichotomous spermatogenesis refers to the production of two types of sperm, namely, eupyrene (nucleate) and apyrene (anucleate) sperm, which differs in structure and function (Chen et al., 2020). This phenomenon is characteristic of Lepidopteran moths and butterflies. In *S. cerealella* males, germ cells develop within cysts formed by somatic cells throughout spermatogenesis. Each cyst contains either eupyrene or apyrene spermatocytes, which undergo two rounds of meiosis to form spermatids. These spermatids then elongate and differentiate into sperm bundles. Eupyrene sperm bundles are characterized by needle-shaped nuclei in the front part of the elongating cells and contain haploid sperm. On the other hand, apyrene sperm bundles are shorter and lack any nuclear material. Eupyrene sperms are responsible for fertilizing eggs, while apyrene sperms serve several functions, such as assisting in eupyrene sperm transfer and motility, reducing sperm competition, and providing nourishment for eupyrene sperm (Chen et al., 2020). The presence of Siah homologs in other insect species suggests a conserved function in regulating developmental processes. For example, Siah proteins have been implicated in the regulation of Wnt/β-catenin signaling pathways, which are crucial for various developmental events, including those related to reproductive biology (Ji et al., 2017). Additionally, comparative studies indicate that Siah proteins may exhibit distinct expression patterns and regulatory mechanisms in different species, which could provide insights into their specific roles in spermatogenesis and other physiological processes (Kang et al., 2014).

Here we used the toolkit of *Sitotroga cerealella*, a destructive stored product pest which possess dichotomous spermatogenesis, and reveal the role of *ScE3 Siah* in dichotomous spermatogenesis. We show that knockdown of *ScE3 Siah* through RNAi caused dichotomous sperm impairments and abnormal spermatophore formation. Our results revealed the novel role of *ScE3 Siah* in dichotomous spermatogenesis and infertility of hexapods.

## Material and methods

### Tested insects

The *S. cerealella* strain related to wheat grain was reared in a laboratory under controlled conditions: temperature maintained at 28°C ± 1°C, relative humidity at 75% ± 5%, and a photoperiod of 14:10 h (light:dark). The insects were reared in circular, transparent glass bottles with dimensions of 10 cm in height and a radius of 4 cm. Pupae-containing grains were carefully chosen and transferred to glass tubes for further rearing. Newly emerged adult moths were used to differentiate between males and females for experiments.

### RNA extraction and cDNA synthesis

Various tissues such as testes, ejaculatory ducts, head, thorax, and fat body, were carefully dissected from adult moths. These tissues were then rinsed with phosphate-buffered saline and rapidly cooled using liquid nitrogen to facilitate RNA extraction. Extraction was carried out utilizing TRIzol reagent (Invitrogen, United Status) following the company’s guidelines. The quality and concentration were evaluated using a Nanodrop spectrophotometer (Thermo Fisher, MA, United Status), while RNA integrity was confirmed on a 1.2% agarose gel. cDNA synthesis was performed using a one-step gDNA removal and cDNA synthesis SuperMix, as per recommended procedures, with 500 ng of RNA template.

### Sequence analysis and tissue specific expression analysis

The sequence encoding E3 ligase *Siah* was identified from the testis-specific transcriptome of *S. cerealella*. Bioinformatic tools such as SMART (http://smart.embl.de/), Pfam (http://pfam.xfam.org/) and NCBI (http://www.ncbi.nlm.nih.gov/) were employed to found the conserved domains in the protein sequence of *E3 Siah*.

The expression level of tissue-specific *ScE3 Siah* mRNA was quantified using quantitative real-time PCR (qRT-PCR) conducted on a Bio-Rad iQ2 thermocycler (Bio-Rad, United Status). The cDNA underwent a 1:10 dilution, with 2 μL then applied to a 20 μL reaction mixture comprising 10 μL of SYBR Green Master Mix from Thermo Fisher Scientific (United Status), 0.8 μL each of sense and antisense primers, and 6.4 μL of sterilized ultra-pure water. Three biological replicates were performed, with three technical replicates for each biological sample. The qRT-PCR reaction protocol involved an initial denaturation stage at 95°C for 30 s, followed by 40 cycles comprising denaturation at 95°C for 5 s and annealing/extension at 60°C for 30 s. A melting curve analysis was performed at the end of each reaction. The expression levels were normalized using glyceraldehyde-3-phosphate dehydrogenase (GAPDH) as an internal control. Gene-specific primers were designed using Geneious v.10.1.2, and their sequences are provided in Supplementary Table 1. A standard curve was generated to verify primer specificity and efficiency.

### Double stranded (dsRNA) invitro synthesis

The dsRNA was synthesized *invitro* utilizing the T7 RiboMAX™ Express kit (Promega, United Status) according to the manufacturer’s guidelines. The cDNA template was derived from isolated RNA employing TRIzol Reagent (Invitrogen Life Technologies, United Status) from *S. cerealella* testis. The target genes, *ScE3 Siah* and *GFP* (Green fluorescent protein), underwent amplification using *T7GFP* and *T7 ScE3 Siah* primer sets (Supplementary Table 1), resulting in the generation of sense and anti-sense strands individually. The thermal cycling conditions were set as follows: An initial denaturation at 95°C for 5 min, with subsequent 30 cycles comprising denaturation at 95°C for 30 s, annealing at 50°C for 30 s, and extension at 72°C for 30 s, concluding with a final extension at 72°C for 10 min. The products were visualized via 1% Agarose gel electrophoresis before purification through ethanol precipitation, and quantified utilizing a UV–visible spectrophotometer. PCR products purified to a concentration of 1 μg/μL were transcribed to yield single-stranded RNAs. Equal volumes of single-stranded RNAs were subjected to annealing through incubation at 70°C for 10 min to produce double-stranded RNA. The dsRNA underwent further purification following the protocol outlined by RiboMAX Express (Promega, United Status), and then quantification via spectrophotometer. Stocks of dsRNA were prepared to a final concentration of 1 μg/μL dsRNA corresponding to the target genes (*ScE3 Siah* and *GFP* as a control) was injected into the newly emerged adult male moths. The injection was performed using a fine glass capillary needle, ensuring that the dsRNA was delivered directly into the hemocoel. The injection site was the fifth–sixth internode membrane of the abdomen. The males injected with dsRNA were placed in an artificial climate incubator at a temperature of 28 ± 1°C, a relative humidity of 75 ± 5%, and a photoperiod of L:D = 14:10.

### Light microscopy of testes

Testes from 15 adult moths were collected and dissected into ds*GFP* and ds*ScE3 Siah* groups in each replicate. They were fixed in aqueous Bouin’s solution for 8 h, followed by dehydration in alcohol, clearing in xylene, embedding in histological paraffin, and slicing into 3 μm thick sections using a Leica RM 2250 microtome. These sections were stained with hematoxylin and eosin (H&E). Observation was recorded using a NIKON Eclipse Ti-SR microscope, and digital images were captured with a digital camera.

### Transmission electron microscopy

The testes of 15 adult moths from each replicate of the ds*GFP* and ds*ScE3 Siah* groups were collected and immersed in a fixative solution comprising 2.5% glutaraldehyde and 4% paraformaldehyde in 0.1M phosphate buffer (pH 7.3) for 24 h at room temperature (RT). They were treated with 1% osmium tetroxide in the same buffer for 2 h for post-fixation. After fixation, the specimens were washed with distilled water and stained with a 0.5% aqueous solution of uranyl acetate for 2 h. Dehydration was carried out using a gradient of acetone concentrations (50%, 70%, 90%, and 100%), followed by embedding in Araldite^®^ resin. Ultrathin sections were prepared and stained with a saturated alcoholic solution of uranyl acetate and lead citrate before examination with a JEOL-1200E transmission electron microscope (TEM). Image acquisition was performed using a MORADA-G2 TEM camera.

### Sperm motility analysis

Sperm motility and flagellar characteristics, including wavelength, velocity, amplitude, and frequency, were assessed using a Nikon Ti Microscope at either 10x or ×20 magnification. Recording was performed with NIS-Elements Viewer software and a Nikon DS-Ri2 color camera. Image analysis was conducted using ImageJ and FIJI software, along with relevant plugin tools. Sperm velocity was determined by tracking undulating waves and measuring the speed of these waves by clicking on the peak of each wave in every frame utilizing the Manual Tracking Plugin in ImageJ (available at https://imagej.nih.gov/ij/plugins/track/track.html). The instantaneous velocity for each wave was averaged. Amplitude measurements were taken from the approximate midpoint of the wave, from the middle peak to the trough, utilizing the line tool at a 0-degree angle. Wavelength measurements were obtained using the line tool to measure from peak-to-peak or trough-to-trough, depending on the clarity of the wave and ease of measurement. Frequency assessment involved visually estimating a complete cycle of the waveform by selecting a frame within the stack and then identifying where the waveform repeated throughout the stack.

### Oviposition assay and spermatophore morphological analysis

One day post dsRNA injection, males were paired with virgin females emerging within the last 12 h to monitor mating behaviors. Egg-laying paper was placed into the mating tube, and egg counts were recorded after 5 days. Males injected with ds*GFP* served as controls, with 20 pairs assessed across three replicates. After housing males injected with ds*ScE3 Siah* and ds*GFP* in an artificial climate incubator for a day, they were separated and paired with virgin females. Mating behaviors were observed, followed by immediate dissection of females to examine and measure spermatophore morphology under a microscope. Twenty spermatophores were analyzed per group, with three biological replicates. Images were captured using LightTools software.

### Sperm counts in multiple male reproductive tissues

For sperm counts, the method was adopted as previously explained in our work (Yan et al., 2022). Briefly, testis and ejaculatory ducts from ds*ScE3 Siah* and ds*GFP* injected moths were dissected, cleaned and put in PBS buffer. The dissected tissues were then washed and transferred into PBS buffer. The tissues were gently homogenized, and the resulting mixture was diluted to a final volume of 80 µL with PBS. After the addition of 20 µL of pre-cooled DAPI solution, the mixtures were incubated in the dark at RT for 10–15 min. A small aliquot (2 µL) of the stained solution was then pipetted onto a glass slide and examined using an inverted fluorescence microscope (OLYMPUS, Tokyo, Japan). Sperm cells were identified as fluorescent entities, and their numbers were counted. Each sample was prepared in duplicate using reproductive organs from five individuals per group. Three independent replicates were performed for each experimental group, with male moths injected with dsGFP serving as the control.

### Fluorescent staining of dichotomous sperm bundles

Testes dissected from male moths injected with ds*ScE3 Siah* and ds*GFP* were placed onto a glass slide immersed in PBS, followed by fixation using a solution containing 4% paraformaldehyde (Beyotime, China) in PBS for 1 h. After fixation, the samples underwent three washes with PBS, each lasting for 5 min, then Actin proteins were stained with TRITC Phalloidin (YEASEN, China) for 1 h, while nuclei were stained with Hoechst for 10 min. Afterward, the samples underwent three washes with PBS, were smeared onto a microslide, and observed under the microscope (Olympus, BX53).

### Determination of ATP contents in sperm

The ATP concentration was determined using a luciferase-based ATP bioluminescence assay ATPlite kit (Perkin Elmer, United States) following the manufacturer’s protocol. Mammalian cell lysis solution (50 µL) was mixed with sperm cell suspension (100 µL) from ds*GFP* and ds*ScE3 Siah* injected adult males in each well of a microplate. Substrate solution (50 µL) was added to each well, and the plate was shaken and incubated in darkness. Luminescence was measured with an Envision multimode plate reader (Perkin Elmer United Status). The experiment was triplicated with biological replicates, utilizing a total of 10 insects from each group.

### Statistical analysis

Data analyses were conducted utilizing GraphPad Software, version 6.0 (GraphPad Software Inc., United Status), expressing the data as mean ± standard error mean (SEM). Statistical comparison between different groups were made using Student’s t-test and one-way analysis of variance (ANOVA) with significance set at *p* < 0.05.

## Results

### *ScE3 Siah* is essential for egg production in *S. cerealella*

**FIGURE 1:**
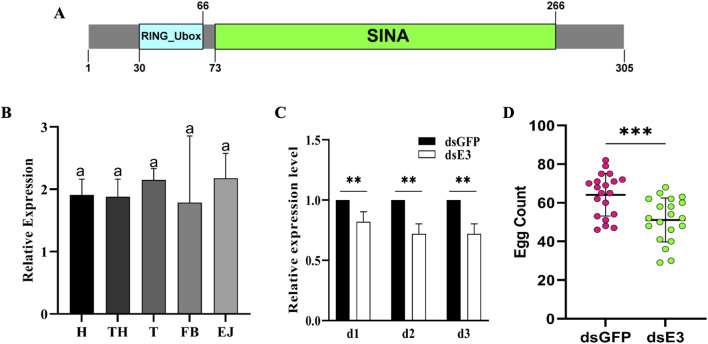
**(A)** Bioinformatics analysis of *Sitotroga cerealella* E3 ligase *Siah.* The sequence showed *RING* and *SINA* conserved domains. **(B)** Relative expression analysis of *ScE3 Siah* in various tissues. GAPDH was used as an internal control. Labels: H = head, TH = thorax, T = testis, FB = fat bodies, and EJ = ejaculatory ducts. Error bars indicate SEM and similar letters indicate non-significant differences compared to the control condition, as determined by one-way ANOVA (*p* > 0.05) **(C)** Normalized expression assay of *ScE3 Siah*. Day-1, day-2 and day-3, post-injection. Error bars indicate SEM, (t-test), **p < 0.01. **(D)** The mean number of eggs laid by female mated with ds*ScE3 Siah* and ds*GFP* male moths. Error bars indicate SEM and asterisk indicates a significant difference (*p* < 0.05, t-test).

The *S. cerealella ScE3 Siah* protein sequence comprises 305 amino acids and exhibits significant similarity to other insect *E3 Siah* proteins. It possesses conserved domains referred to as RING (*Really Interesting New Gene*) and SINA (*Seven in Absentia*) (Figure 1A). Furthermore, investigating the expression pattern of the *ScE3 Siah* in various adult tissues using qRT-PCR, we found widespread and relatively uniform expression across the spectrum of adult tissues including head, thorax, testis, fat bodies, and ejaculatory ducts (Figure 1B).

To clarify the biological function of *ScE3 Siah* in adult male moth reproduction, dsRNA was synthesized specifically designed to suppress *ScE3 Siah* gene expression. *ScE3 Siah* expression level were assessed via qRT-PCR from day 1 to day 3 after microinjection (Figure 1C). The results showed a significant reduction in *ScE3 Siah* expression within 3 days compared to the control group injected with ds*GFP*, indicating successful utilization of RNAi to silence the expression of *ScE3 Siah* in male moths (Figure 1C).

Male moths injected with ds*ScE3 Siah* displayed normal behavior and successfully mated with female partners, exhibiting mating behavior indistinguishable from that of ds*GFP* moths. However, females mating with ds*ScE3 Siah-*injected males laid significantly fewer eggs compared to those that mating with ds*GFP-*injected males (Figure 1D). Over 5 days, females mated with ds*GFP*-injected males laid an average of 61 eggs, while those mated with ds*ScE3 Siah*-injected males laid only 51 eggs (Figure 1A). These results highlight *ScE3 Siah* crucial role in male fertility, directly impacting moth reproductive success.

### *ScE3 Siah* knockdown caused testis and sperm morphology disruption of *S. cerealella*

**FIGURE 2:**
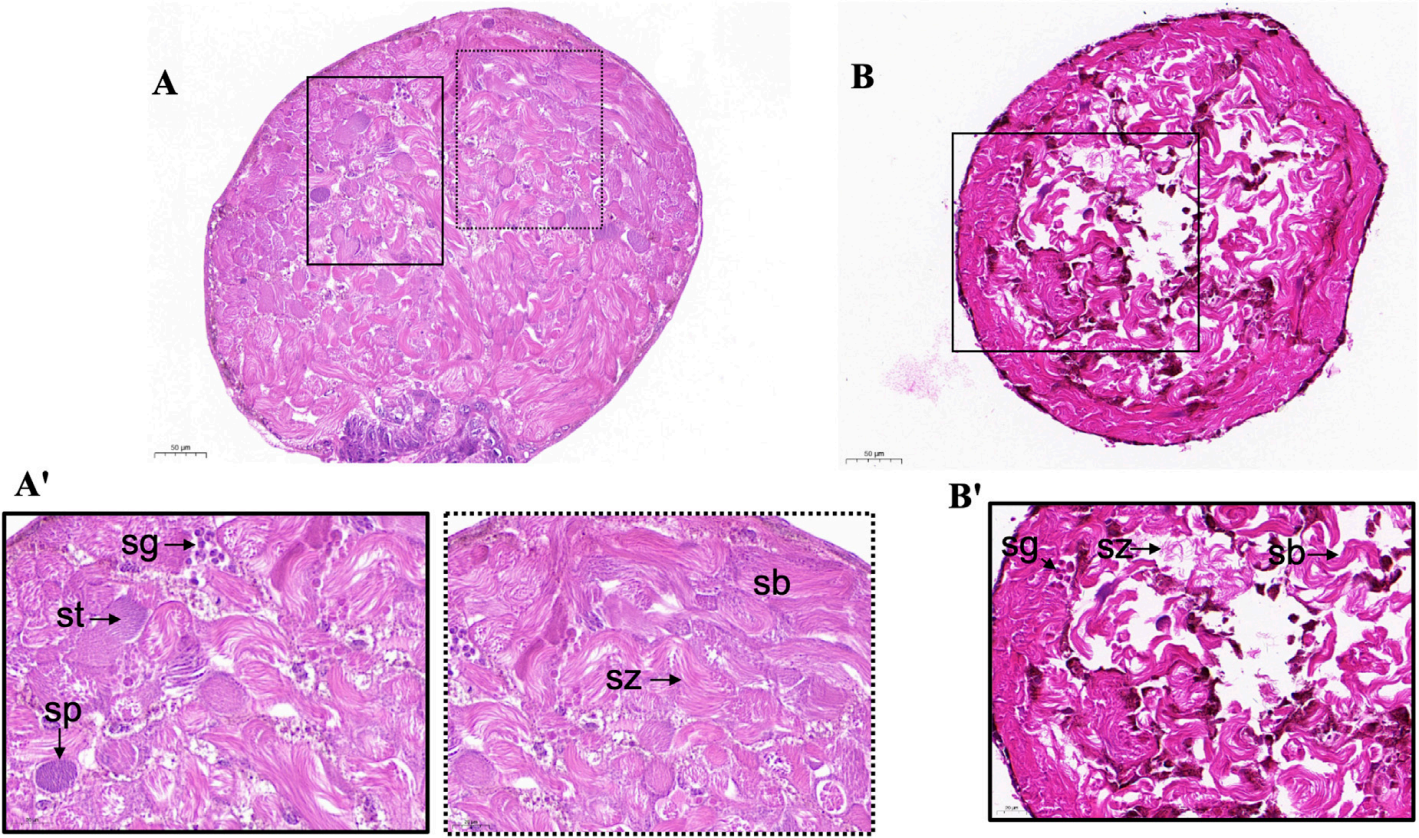
Light microscopy of adult *S. cerealella* testes. **(A, A')** Representative images of light microscope of dsGFP adults moth testes. The testes were collected from virgin adults and stained with Hematoxylin and eosin. The sections indicate spermatogonia (Sg), spermatocytes (Sp), spermatids (St), spermatozoa (Sz) and normal sperm bundles (Sb) and cyst distribution. Enlargement of A' show the morphology and distribution of different stages of sperm cells, spiral structure of sperm bundle, loosely arranged spermatozoa. **(B)** Representative images of histological sections from adult testes of *dsScE3 Siah*. Histological sections indicate disruption in testes morphology. Improper and irregular distribution of spermatozoa indicate in all sections. Histological sections indicate ruptured and cleave sperm bundles with empty spaces. Scale bars, 50 μm **(A, B)**, 20 μm **(A', B')**.

We examined the morphology of the testes of adult male moths microinjected with ds*ScE3* and ds*GFP*. Histological examination via paraffin section and hematoxylin eosin staining revealed that the structure and morphology of the testes in male moths injected with ds*GFP* exhibited normal sperm morphology and a well-organized distribution of cysts and sperm bundles. Spermatozoa were loosely bundled and displayed a filamentous spiral arrangement (Figure 2A'). In contrast, the ds*E3 Siah* phenotypes led to significant alterations in the shape and morphology of the adult testes. These testes exhibited notable signs of cellular stress, with disrupted distribution and organization of spermatocytes, spermatids, cysts, and sperm bundles, ultimately affecting sperm numbers (Figure 2B').

Taken together, these observations reveal that ds*E3 Siah* is essential for spermatogenesis, providing evidence of impaired dichotomous spermatogenesis.

### *ScE3 Siah* knock down cause sperm defects in adult *S. cerealella*

**FIGURE 3:**
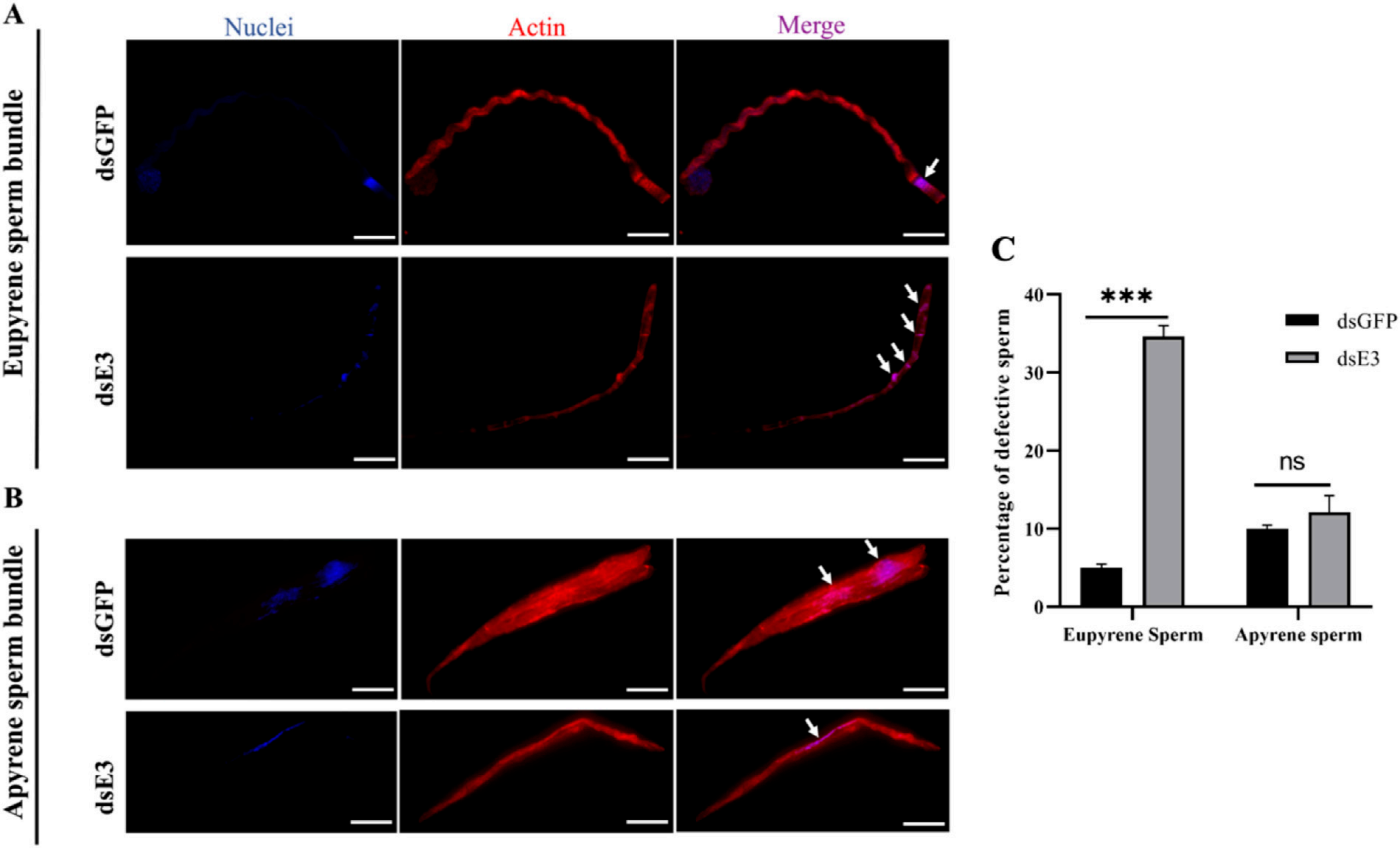
Representative images illustrating dichotomous sperm bundles **(A)** Eupyrene sperm bundles **(B)** Apyrene sperm bundles obtained from ds*GFP* (top) and ds*E3* (bottom) samples collected from adult male moths. Nuclei were visualized using Hoechst staining, while TRITC-Phalloidin staining was employed to visualize actin, demonstrating the structure of the sperm bundles. White arrows indicate the nuclei. Scale bar represents 25 μm. **(C)** Quantification of the defective sperm percentage of eupyrene and apyrene sperm in dsGFP and dsE3 Siah groups. Error bars indicate SEM and asterisk indicates a significant difference (*p* < 0.05, t-test), ns = non-significant.

We then employed fluorescence staining with phalloidin and Hoechst to examine the structure of dichotomous sperm bundles in the adult testes of *S. cerealella*. Males injected with ds*GFP*, the eupyrene sperm displayed a thick, brush-like head with neatly arranged nuclei near the head (Figure 3A, ds*GFP*). However, in males injected with ds*ScE3 Siah*, abnormalities in eupyrene sperm were evident, with nuclei showing a diffuse distribution within the sperm bundles (Figure 3A, ds*E3*). Additionally, a disrupted cytoskeleton was observed, with hollow bundles featuring distinctive grooves at the various points along the bundles (Figure 3A, ds*E3*). The helical structure of the sperm bundles was disrupted in ds*ScE3 Siah* phenotypes.

For apyrene sperm bundles, those from ds*GFP*-injected male moth testes exhibited a slender morphology with nuclei irregularly distributed near the bundle’s center (Figure 3B, ds*GFP*). No notable differences were observed in the apyrene sperm bundles between the two groups, however, some intermediate changes in the structure of apyrene sperm has been observed (Figure 3B, ds*E3*).

### Impact of *ScE3 Siah* knockdown on sperm ultrastructure

**FIGURE 4:**
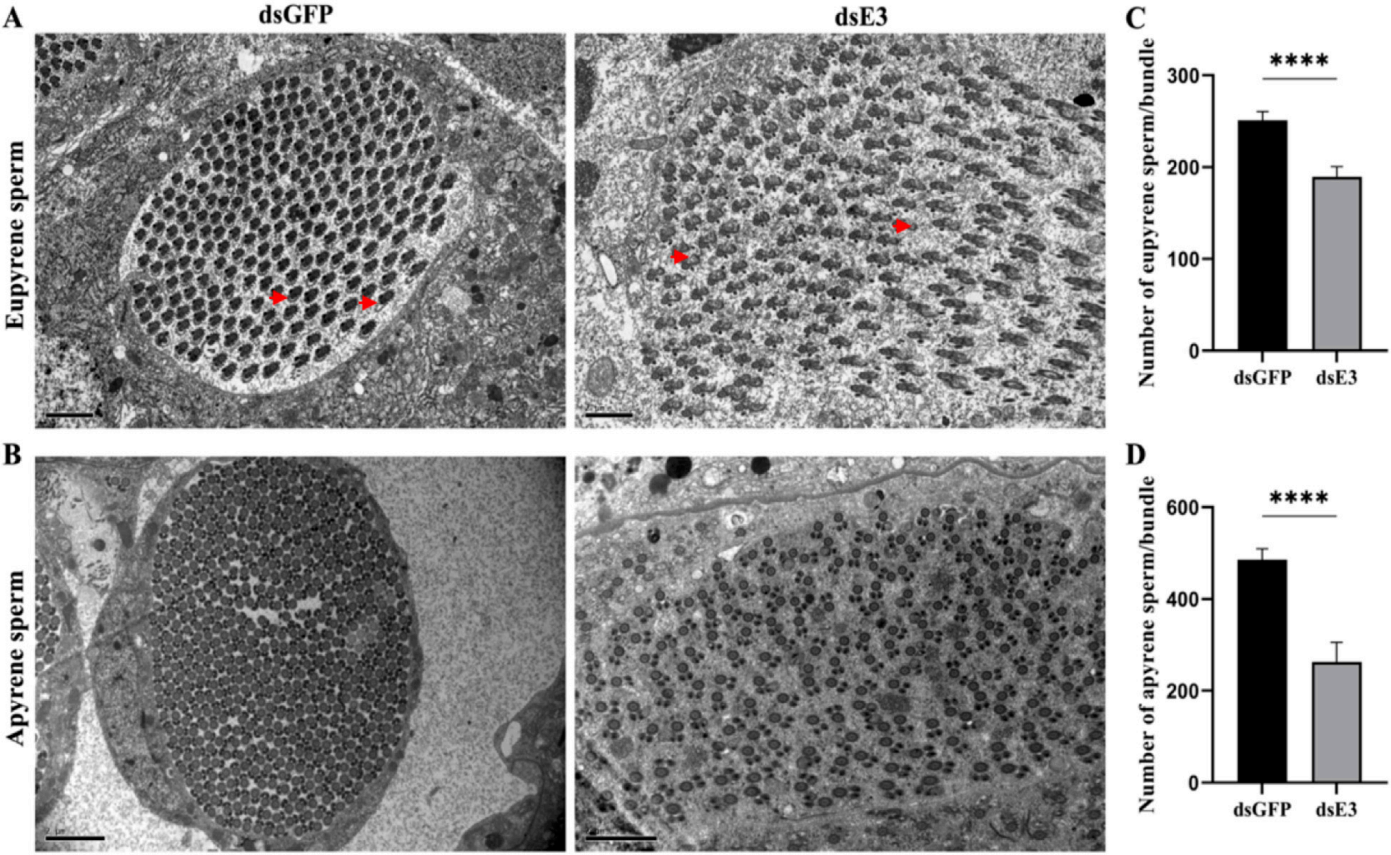
Internal ultra-structures of sperm bundles visualized by transmission electron microscopy of ds*GFP* and ds*E3* groups. **(A)** Representative images of the internal structures of the eupyrene sperm bundles from ds*GFP* (left) and ds*E3* (right) adult male moths. Scale bars, 2 μm. **(B)** Representative images of the internal structures of the apyrene sperm bundles from ds*GFP* (left) and ds*E3* (right) from adult male moths. Scale bars, 2 μm **(C)** Statistical quantification of eupyrene spermatozoa in each bundle. **(D)** Statistical quantification of apyrene spermatozoa in each bundle. Error bars indicate SEM and (t-test), ****p < 0.0001.

To further investigate the role of *ScE3 Siah* in the structural changes of dichotomous sperm, we used transmission electron microscopy (TEM) to analyze the ultrastructural alterations in apyrene and eupyrene sperm in the testes of male moths injected with ds*GFP* and ds*ScE3 Siah*. Eupyrene sperm have a distinct head and flagellum. The head contains the nucleus, while the flagellum is structured with a 9 + 9 + 2 microtubule arrangement, along with mitochondrial derivatives. Additionally, plasma membrane extensions form reticular and lacinate appendages on both the head and flagellum. On the other hand, apyrene sperm lack a nucleus but still have a flagellum with the same 9 + 9 + 2 microtubule structure. The flagellum is associated with two mitochondrial derivatives that run parallel to the axoneme. We observed disrupted and defective ultrastructure in the eupyrene sperm of ds*ScE3 Siah* injected male testes (Figure 4A, ds*E3*) compared to those of ds*GFP* injected males, which exhibited normal axonemes and mitochondrial derivatives in eupyrene sperm (Figure 4A, ds*GFP*). Conversely, no obvious changes were observed in the structure of apyrene sperm between the ds*GFP* and ds*ScE3 Siah* phenotypes (Figure 4B). However, we did observe a significant decrease in the number of both apyrene and eupyrene sperm within bundles in the ds*ScE3 Siah* phenotypes.

The mean number of eupyrene sperm per bundle was significantly reduced in ds*ScE3 Siah* injected male moths compared to ds*GFP* injected males, with counts of 189 and 254, respectively (Figure 4C). Similarly, the mean number of apyrene sperm per bundle was significantly lower in the ds*ScE3 Siah* phenotypes, with 262 sperm counted, compared to 485 sperm in the ds*GFP* phenotypes (Figure 4D). These findings indicate that *ScE3 Siah* leads to a significant reduction in the number of dichotomous sperm and induces aberrant morphological and structural changes specifically in eupyrene sperm.

### *ScE3 Siah* knockdown causes abnormal spermatophore formation and a reduction in dichotomous sperm number

As we observed abnormalities and defective structures in dichotomous sperm within the testes of male moths following *ScE3 Siah* knockdown, we proceeded to evaluate the process of sperm migration from the testes to females. Male moths injected with ds*ScE3 Siah* and ds*GFP* were mated with normal females, and upon completion of mating, the females were dissected to examine their spermatophores.

**FIGURE 5:**
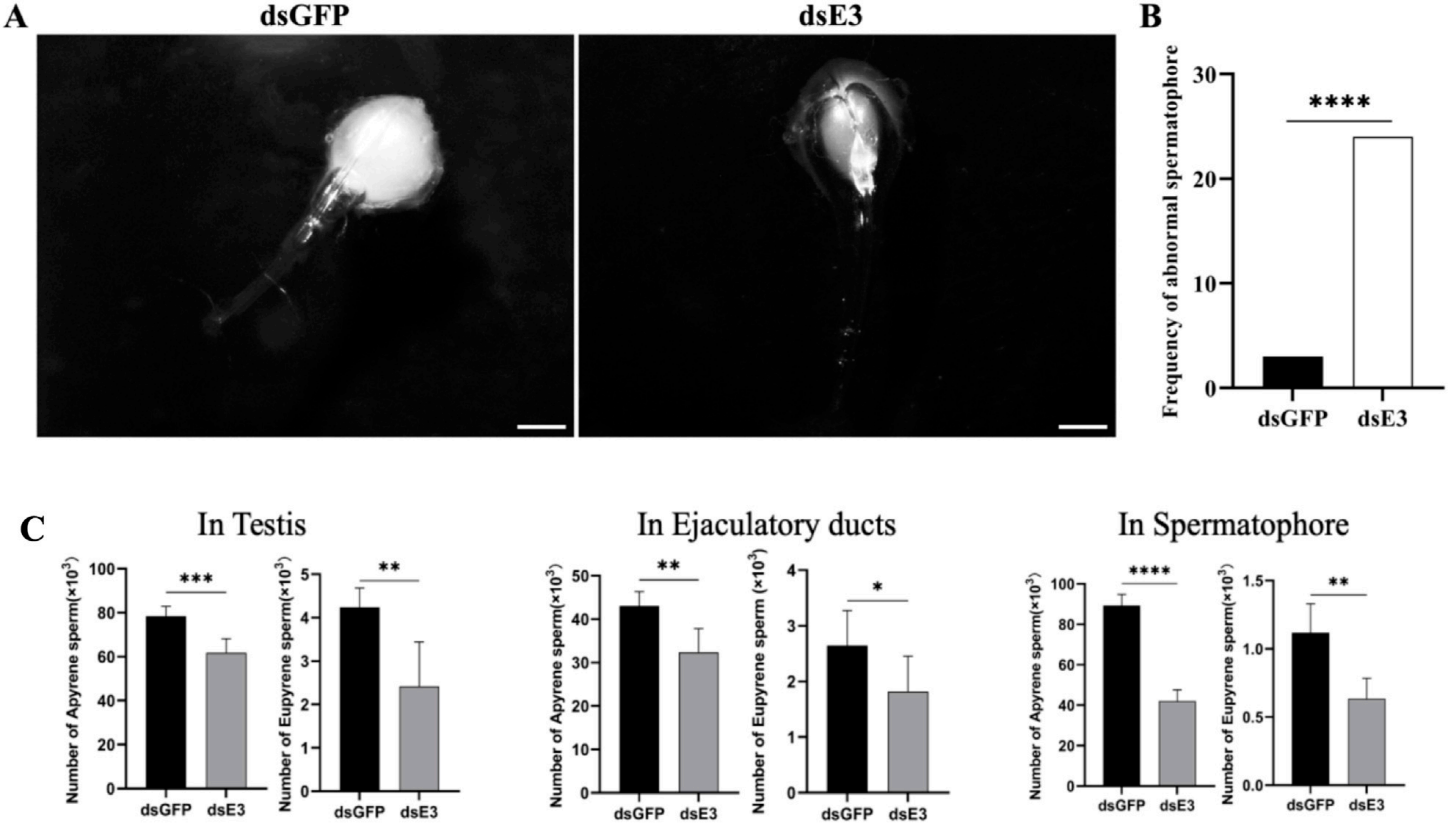
Representative images of the spermatophores **(A)** ds*GFP* (left panel) and ds*ScE3 Siah* (Right panel) post-injection. Scale bars, 1 mm **(B)** Statistical analysis of the frequency of abnormal spermatophore formation between ds*GFP* and ds*ScE3 Siah* group. The statistical analysis was performed using the chi-square Fisher exact test. Asterisks denote significance levels, with **** indicating *p* < 0.0001. **(C)** Sperm quantification analysis of ds*GFP* and ds*E3* phenotypes. Error bars indicates SEM, (t-test), ****p < 0.0001, ***p < 0.001, **p < 0.01, *p < 0.05.

We observed that females mated with ds*GFP* injected males had normal and filled spermatophores, indicating successful sperm transfer (Figure 5A, ds*GFP*). In contrast, females mated with males injected with ds*ScE3 Siah* exhibited empty and abnormal spermatophores, with a significant reduction in the number of sperm (Figure 5A, ds*E3*). A total of 30 females from each group were dissected after mating, and the frequency of abnormal spermatophores in the ds*ScE3 Siah* injected group was significantly higher than that in the ds*GFP* group (Figure 5B). Among 30 females mated with ds*ScE3 Siah* injected males exhibited 24 abnormal spermatophore, whereas female mated with ds*GFP* injected male had 3 abnormal spermatophore (Figure 5B). These findings reveal that *ScE3 Siah* knockdown results in defects in the sperm transfer process from male to female moths.

Upon observing morphological changes of the spermatophores and considering the significance of sperm in moth reproduction, we conducted a quantitative analysis of dichotomous sperm in various tissues of male moths injected with ds*GFP* and ds*ScE3 Siah*, as well as in mated females. Our findings revealed a significant reduction in the number of both apyrene and eupyrene sperm in the testes, ejaculatory ducts, and spermatophores of moths injected with ds*ScE3 Siah* compared to those injected with ds*GFP* (Figure 5C). In the testes, the number of apyrene sperm decreased significantly from 78.33 × 10^3^ in the ds*GFP* group to 61.83 × 10^3^ in the ds*ScE3 Siah* group (*p* < 0.001). Similarly, the eupyrene sperm count decreased from 4.23 × 10^3^ to 2.41 × 10^3^ (*p* < 0.01) in the ds*GFP* and ds*ScE3 Siah* groups, respectively. In the ejaculatory ducts, the apyrene sperm count decreased from 43 × 10^3^ in the ds*GFP* group to 32.33 × 10^3^ in the ds*ScE3 Siah* group, while the eupyrene sperm count decreased from 2.65 × 10^3^ to 1.81 × 10^3^ (*p* < 0.05) in the respective groups.

Analysis of spermatophores from females mated with ds*ScE3 Siah* males revealed a significant reduction in both apyrene and eupyrene sperm numbers compared to those mated with ds*GFP* males. The apyrene sperm count decreased from 89.37 × 10^3^ to 42 × 10^3^ (*p* < 0.0001), while the eupyrene sperm count decreased from 1.117 × 10^3^ to 0.63 × 10^3^ (*p* < 0.01) in females mated with ds*GFP* and ds*ScE3 Siah* males, respectively.

### *ScE3 Siah* knockdown decrease dichotomous sperm motility but not ATP contents

**FIGURE 6:**
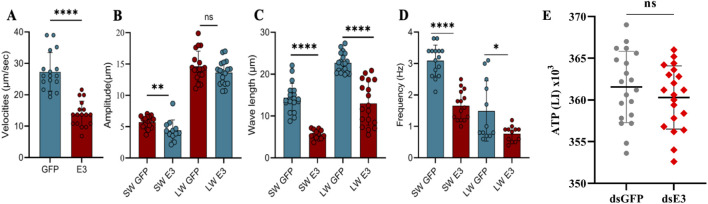
Sperm motility parameters (Velocities **(A)**, amplitude **(B)**, wavelength **(C)** and frequencies **(D)**) were assessed for ds*GFP* and ds*E3* groups. ATP levels were measured in spermatozoa of ds*GFP* and ds*ScE3 Siah* male moths **(E)**. The experiment was triplicated with biological replicates, utilizing a total of 50 male moths from each group. Statistical significance was determined using a t-test, with ****p < 0.0001, ***p < 0.001, **p < 0.01, *p < 0.05. Non-significant results were denoted as ns (*p* > 0.05).

As the knockdown of *ScE3 Siah* resulted in reduced sperm count and structural disruptions in dichotomous sperm, inhibiting their transfer from male to female moths, we conducted further analysis to assess the motility of dichotomous sperm in female reproductive tracts after mating with ds*GFP* and ds*ScE3 Siah* injected males. Our investigation revealed a significant reduction in major sperm motility parameters due to the silencing of *ScE3 Siah* (Figures 6A–D).

Specifically, the knockdown of *ScE3 Siah* led to a substantial decrease in the wave velocities of dichotomous sperm, dropping from 27.30 μm/s to 13.91 μm/s (*p* < 0.0001) compared to the ds*GFP* phenotypes (Figure 6A). Additionally, the ds*ScE3 Siah* resulted in a reduction in the amplitude of short waves (SW) from 5.73 μm to 4.407 μm compared to ds*GFP*, while the amplitude of long waves (LW) remained relatively unchanged, with values of 14.64 μm for ds*GFP* and 13.62 μm for ds*ScE3 Siah* (*p* > 0.05) (Figure 6B).

Moreover, in the ds*ScE3 Siah* group, both the wavelengths of SW and LW exhibited a decrease compared to the ds*GFP* control group (Figure 6C). Specifically, the average wavelength of SW decreased from 14.38 μm in ds*GFP* to 5.63 μm in ds*ScE3 Siah* (*p* < 0.0001), while for LW, the wavelength decreased from 22.68 μm in ds*GFP* to 13.02 μm in ds*ScE3 Siah* (*p* < 0.0001).

The frequencies of both SW and LW were significantly reduced in the ds*ScE3 Siah* group (Figure 6D). The frequency of SW decreased from 3.09 Hz in ds*GFP* to 1.66 Hz in ds*ScE3 Siah* (*p* < 0.0001), whereas for LW, the frequency decreased from 1.49 Hz in ds*GFP* to 0.76 Hz in ds*ScE3 Siah* (*p* < 0.0001).

Since the silencing of *ScE3 Siah* caused decreased in sperm motility parameters and adenosine triphosphate (ATP) is considered the major source for metabolic functioning of sperm and related activities, we conducted measurements of sperm ATP content. Surprisingly, our analysis revealed that silencing *ScE3 Siah* had no apparent impact on the profiling of sperm ATP content (Figure 6E).

## Discussion

The ubiquitin system, particularly E3 ubiquitin ligases, has been extensively studied across various biological processes, including autophagy, immune responses, and disease therapies (Yin et al., 2020; Morrow et al., 2015). These studies highlight the diverse roles of E3 ligases in cellular functions and disease pathways. For instance, the involvement of ubiquitin in mediating autophagy highlights the participation of E3 ligases in maintaining cellular homeostasis (Yin et al., 2020; Morrow et al., 2015).

Here we report the identification of the *S. cerealella* E3 ligase, *ScE3 Siah*, comprising 305 amino acids, which shares significant similarity with other insect E3 *Siah* proteins and harbors conserved RING (*Really Interesting New Gene*) and SINA (*Seven in Absentia*) domains. *ScE3 Siah* exhibits widespread and relatively uniform expression across various adult tissues, aligning with the fundamental role of E3 ligases in regulating essential cellular processes. The similarity of *ScE3 Siah* to other insect *E3 Siah* proteins suggests a conserved function in ubiquitin-mediated pathways.

This study provides significant insights into the impact of *ScE3 Siah* knockdown on the reproductive physiology of *S. cerealella*. The findings reveal that knockdown of *ScE3 Siah* results in disruptions in testes and sperm morphology. Additionally, abnormalities in the structure of dichotomous sperm bundles were observed, along with defective ultrastructure in eupyrene sperm, ultimately leading to a reduction in the number of dichotomous sperms. These ultrastructural changes in sperm can significantly affect their overall motility and fertilization ability. Reduced mitochondrial function, altered membrane composition, impaired acrosome structure and enzyme activity, and disrupted motility mechanisms all contribute to decreased sperm quality and fertility (Lopata et al., 2020). In Lepidopteran insects, dichotomous spermatogenesis produces eupyrene and apyrene spermatozoa and is controlled by sophisticated network of genes. Several genes, such as *Prmt5-Vasa* module and *Bm-Sxl*, have been implicated in spermatogenesis along with genes involved in spermatogenesis identified through comprehensive functional enrichment analysis (Yan et al., 2022; Lopata et al., 2020; Yang et al., 2023; Sakai et al., 2019; Yang et al., 2022). Additionally, epigenetic regulation, such as histone modifications, and the involvement of miRNAs and piRNAs have been found to play roles in spermatogenesis (Qian et al., 2023). Moreover, stress response genes, including heat shock proteins and DNA repair genes, are expressed during spermatogenesis (Yuen et al., 2014).

*ScE3 Siah* knockdown significantly reduces dichotomous sperm motility, emphasizing its role in regulating sperm function. Studies have suggested that E3 ligases, such as *March7*, *March10*, and *CUL4*, can be crucial factors in spermatogenesis and fertility by modifying germ cell chromatin condensation, alignment, and/or crossover (Yuen et al., 2014; Aguilar-Mahecha et al., 2001; Zhao et al., 2013; Lin et al., 2016; Yin et al., 2016; Iyengar et al., 2011; Gvakharia et al., 2003). A mutation in the E3 ligase *Siah1a* in mice has been found to result in sub-fertility in females and sterility in males due to spermatogenesis blockage. The research indicates that *Siah1a* is essential for normal progression beyond metaphase I, hinting at its involvement in a novel E3 complex functioning late in the first meiotic division (Whittington et al., 2015). E3 ligases maintain the structural integrity of developing spermatozoa (Lin et al., 2016). For example, the knockout of *CUL4B* in mice results in male infertility because it disrupts the preservation of the spermatogonial stem cell environment (Yin et al., 2016).

*ScE3 Siah* knockdown inhibits the transfer and motility of dichotomous sperm in *S. cerealella*. The abnormal spermatophore formation in females mated with *dsScE3 Siah* males, along with the reduced dichotomous sperm number and motility, as well as unaffected sperm ATP content, provides valuable insights into the transcriptional changes that may influence sperm development and function in moths. This suggests that *ScE3 Siah* might have pivotal function in the control of sperm transfer and storage. In Cotton leaf worms *Spodoptera littoralis*, the discharge of sperm bundles from testes was inhibited by the reduction of F-actin, implicating actin in the initiation of dichotomous sperm discharge (Gvakharia et al., 2003). We found that knockdown of *ScE3 Siah* also caused disruption of actin structure, as evident by fluorescence microscopy. The actin cytoskeleton plays a crucial role in various sperm functions, including the acrosome reaction, capacitation, and motility. Several studies have highlighted the importance of actin-regulating proteins in these processes (Sosnik et al., 2010; Romarowski et al., 2015; Salgado-Lucio et al., 2020). One potential mechanism by which *ScE3 Siah* may influence the actin cytoskeleton is through the ubiquitination and regulation of actin-binding proteins. For example, the actin-capping protein CAPZA3 has been shown to be involved in the control of actin polymerization during spermiogenesis and the acrosome reaction (Sosnik et al., 2010). *ScE3 Siah* may potentially regulate the ubiquitination and degradation of CAPZA3, thereby affecting actin dynamics and sperm release and transfer. Similarly, the LIMK1/Cofilin signaling pathway, which is essential for actin polymerization and the acrosome reaction, could be a target of *ScE3 Siah*-mediated ubiquitination (Romarowski et al., 2015). Furthermore, focal adhesion kinase (FAK) has been reported to regulate actin polymerization during sperm capacitation via the ERK2/GEF-H1/RhoA signaling pathway (Salgado-Lucio et al., 2020). *ScE3 Siah* may potentially interact with and modulate the ubiquitination of FAK or other components of this pathway, thereby affecting actin dynamics and sperm function. However, the unaffected sperm ATP content upon *ScE3 Siah* knockdown suggests that the regulation of ATP content in sperm may involve different molecular pathways than those affected by *ScE3 Siah* knockdown. One potential mechanism could involve the regulation of glycolytic enzymes or mitochondrial function by *ScE3 Siah*. Studies have shown that sperm motility and fertilization ability are closely linked to ATP production, which can be derived from both glycolysis and oxidative phosphorylation (Gong et al., 2017). *ScE3 Siah* may regulate the ubiquitination and stability of key enzymes or regulators involved in these energy-producing pathways, thereby maintaining ATP levels in sperm despite the knockdown of *ScE3 Siah.* Additionally, *ScE3 Siah* may interact with signaling cascades that modulate sperm metabolism and energy homeostasis, such as the AMPK or mTOR pathways (Pal et al., 2020). These pathways are known to integrate various cellular signals and coordinate energy production and utilization. By regulating the activity or stability of components within these pathways, *ScE3 Siah* may be able to maintain ATP levels in sperm, even in the absence of its direct regulation of the actin cytoskeleton.

Collectively, this study demonstrated that the *ScE3 Siah* performs an essential function in spermatogenesis and reproductive processes in *S. cerealella*. Knockdown of *ScE3 Siah* gene expression leads to impaired sperm production, compromised sperm motility, and disruptions in sperm ultrastructure.

## Conclusion

This study employed RNAi technology in conjunction with biochemical and electron microscopy analyses of dichotomous sperm and testes in adult *S. cerealella* to uncover the role of *ScE3 Siah* in dichotomous spermatogenesis. Our findings demonstrate that *ScE3 Siah* is essential for the normal progression of dichotomous spermatogenesis. Knockdown of *ScE3 Siah* results in significant defects in sperm motility and ultrastructure, indicating its crucial involvement in these processes. This study not only offers valuable insights into pest control strategies targeting Lepidopteran pests but also unravels a novel function of *ScE3 Siah* in regulating dichotomous spermatogenesis.

## Data Availability

The raw data supporting the conclusions of this article will be made available by the authors, without undue reservation.
